# Supplementary material for: Patterns of Intron Gain and Loss in Fungi
Source: PLoS Biol. 2004 Nov 30;2(12):e422. doi: 10.1371/journal.pbio.0020422 (PMC532390; doi:10.1371/journal.pbio.0020422)
Supplement: Table S1 — Also available at http://genes.mit.edu/NielsenEtAl/. (4.3 MB ZIP). [file pbio.0020422.st001.zip › NielsenEtAl/html/291.html]

AN2321.1.NCU05884.1.MG02204.1.FG03636.1


```
 CLUSTAL W (1.82) Multiple Sequence Alignments - Introns Inserted


Sequence 1: AN2321.1	470 aa
Sequence 2: NCU05884.1	496 aa
Sequence 3: FG03636.1	603 aa
Sequence 4: MG02204.1	537 aa
Alignment Length: 639 aa
Number Identitical Residues: 217 aa
Alignment Score (without introns) 12213


MG02204.1 	MVESTVP---RQKWWKIQWYSDDDTPEERRFIIKLDMVVVPYAVLAYWVKYIDQANLN~-
NCU05884.1	MAVHHQFDTVKRKWYHIKWFADSDTPRERRLIMKLDLLIVPYAFLAYWIKYIDQANLS~A
FG03636.1 	MPVVEN----KKKWYHVQWYSDTDTPEERKFIAKLDLLIVPYAVIAYWVKYIDQSNLN1-
AN2321.1  	MSTVRE----RRKWYHIKWFADQDTKEERRLILKLDALIVPYAFLAYWVKYVDQANIN1-
          	*         ::**::::*::* ** .**::* *** ::****.:***:**:**:*:.  

MG02204.1 	------------------------------NAYVAGMKEELNFQGNELVQLQTMYTIGAV
NCU05884.1	LFLPAFFQSLLQPIKDSEVQLTPRYFPLTDNAYVAGLKEDLGFHGNELVQLQTLYTVGAV
FG03636.1 	------------------------------NAYVAGLKEDLGFKGNELVQLQTFYIIGAV
AN2321.1  	------------------------------NAYVSGLQEDLGLHGNELVELQTMYTVGAV
          	                              ****:*::*:*.::*****:***:* :***

MG02204.1 	VGMVPFMFLFTYIPMYWSIPFMDIMWGLFTLLQYRAHSFGEMAAYRFLVGFFE0AAFFPA
NCU05884.1	VGQIPFMFLFTYLPMHWTIPFLDIAWGFFTLAQYRANSFGQMAAYRFLIGWFE~AAFYAA
FG03636.1 	TGQIPFFFLLTYIPIHWLLPFLDVAWGVFTLLQYRVTSFAELAAYRFLVGWFE~AAFFPI
AN2321.1  	VGQLPFAYLFTRFPMSWVIPFLDIMWGVFTLLQYRATSFGEMMAYRFFVGWFE~AAFFPG
          	.* :** :*:* :*: * :**:*: **.*** ***. **.:: ****::*:** ***:. 

MG02204.1 	MHYVFG1SWYRGHEIARRGGLFYTGLNLGTLTAGLVAAGASHRLEGVNGLSGWRWMYIIC
NCU05884.1	IHYIFG~SWYRGDEISRRGGVFYTGLTLGTLTAGLIQSGASSRLEGVNGLAGWRWMYIIC
FG03636.1 	MHYLFG~SWYKGNEIARRGGIFYVGLSLGTLTAGLIQAGASARLDGVHGLEGWRWMYIIC
AN2321.1  	MHYIFG1AWYRGDEIARRGGCFYVGLTLGTLTASLIQAGTSARLEGLHGLAGWRWMYIIC
          	:**:** :**:*.**:**** **.**.******.*: :*:* **:*::** *********

MG02204.1 	ALITFPVGILGYFLLPGTIEQPNRWILNDKDIQIGKDRLERNGHVTAG-KFKVGNLKKIL
NCU05884.1	ALVTIPVGILGYFILPGTPENPNKWFLKDEDVQIAKERLARVGHSVVHPKFKLGTLKSLA
FG03636.1 	SLITIPVGLLGFLIIPGTPDQPNRRVLNQHDIDVGEKRLKRAGHQSHG-KMQWVHLKKVL
AN2321.1  	AIITIPIGILGYFILPGTPDKPNRLVINDKDIELSKIRLSRAGHTFQG-KFTLRSLTNVL
          	:::*:*:*:**::::*** ::**: .:::.*:::.: ** * **     *:    *..: 

MG02204.1 	FSPRFWTVVLVDILFWNAGIHKTTGSFLLWIKSLGRYSPAQINELGTIAPALGILYNLVA
NCU05884.1	NNWKFWALLMLDIFFWNGSLNYSAGGYLLWLKSLGRYSQSKVNELGTISPAIGIFYVLLI
FG03636.1 	LSPQFWGIIAVDILFWNAGTHSSSGSFLLWIKSLQRYSKAKVNELGTIAPALGIFYTLFV
AN2321.1  	KNWKFWALLFLDIFFWNGCINTSAGGYLLWLKSLKRYSTSRLNELGAISPALGIFYTLFI
          	 . :** :: :**:***.  : ::*.:***:*** *** :::****:*:**:**:* *. 

MG02204.1 	CFASDLILGPAWAITASSLLNASGLVILVIWNVPEAAMWYGFSTMYWANALSSVLHGWVN
NCU05884.1	CFISDLVVGPAWAITIAHVWNIIGLVILVIWNVPESALWFAFMTTYSSVAMSAVLYGWIN
FG03636.1 	CFASDLVLGPAWAITVSTVWNALGLIILTIWNVPESALWFAYSTIYASVALSSVLHGWVN
AN2321.1  	CFASDLVLGPAWAITLAHIWNIIGLVILVIWNVPESALWFAFITTYSAVSMSSVLYGWVN
          	** ***::******* : : *  **:**.******:*:*:.: * * : ::*:**:**:*

MG02204.1 	TLLRDSPEERSFTLVVITIIAQSSTAWTPLLTFPTVESPRFPKGFSFCLACALALVVATH
NCU05884.1	AIMGKSSAERSFAIVLLNTVAQSTTAWTQLLTFPTKEAPQFKKGYSFVLANAVCLIITAQ
FG03636.1 	TQLRASPAERSFTLVLINAISQSTTAWTPLLVFPTVEGPRFPKGYPFALGCAIGLLIATH
AN2321.1  	EQLRYSPVERSITLVILNTVAQSTTAWTPLLVFKTVEGPRFTKGYSFVLANAICLIALAH
          	  :  *. ***:::*::. ::**:**** **.* * *.*:* **:.* *. *: *:  ::

MG02204.1 	VLDVYLKRQ-~-E2AYKSQVTASGEDAQSDGASSEGTPGAK0MLVHKQT--------~--
NCU05884.1	FLNVALKRS-~-K2VRKLKSLRL------------------~---------------~--
FG03636.1 	ALHLWLKHRD2PQ~VERPDQILSEESGNLTEESTNPIEEGG~VQTHKTVDSNTTSIN2LW
AN2321.1  	FIKYFIARQ-~-E~YVFSDRFRSQAQANPTQQT--------~---------------~--
          	 :.  : :    :     .      ...    :                           

MG02204.1 	----CWWGRYCGSGMLS-----------LS2SKCKLELYLGSKVQVRLLSLGAIAVQQVR
NCU05884.1	------------------------------~-----------------------------
FG03636.1 	GIVPTAAGSHCIVACLSHVLIPDLLYQIEQ~GQAKQTFHLGYRRKDRIRILACIPMRTPH
AN2321.1  	------------------------------~-----------------------------
          	                                                            

MG02204.1 	HGFYYQA----------------------------------------
NCU05884.1	-----------------------------------------------
FG03636.1 	QSTGFEIAEVGYSSKGKPDYSTTIKLFFYSIFSAFMFLCFPIDRKSH
AN2321.1  	-----------------------------------------------
          	
```
